# The Musculoskeletal Advanced Transillumination Technique (MATT): A Descriptive Proof-of-Concept Study of a New Method for the Study of the Iliotibial Tract Tested on Fresh Cadaveric Specimens

**DOI:** 10.3390/jfmk10030327

**Published:** 2025-08-26

**Authors:** Sonia Bédard, Alexandre Bédard, Nathaly Gaudreault, Matteo Izzo, François Vézina

**Affiliations:** 1 Centre de Recherche du CHUS, CIUSSSE de l’Estrie-CHUS, Sherbrooke, QC J1H 5N4, Canada; nathaly.gaudreault@usherbrooke.ca; 2 Faculty of Medicine, Université de Montréal, Québec, QC H3C 3J7, Canada; alexandre.bedard.1@umontreal.ca; 3 École de Réadaptation, Faculty of Medicine and Health Sciences, Université de Sherbrooke, Québec, QC J1H 5N4, Canada; 4 Swiss Surgical Practice, 6900 Lugano, Switzerland; matteo.izzo.md@gmail.com; 5 Department of Surgery, Orthopedic Division, Faculty of Medicine and Health Sciences, Université de Sherbrooke, Québec, QC J1H 5N4, Canada

**Keywords:** iliotibial band, lateral femoral epicondyle, knee biomechanics, tissue displacement, transillumination technique

## Abstract

**Background:** The iliotibial band (ITB) is an anatomically complex structure with multiple proximal and distal attachments, making its mechanical behavior difficult to interpret. In the study of iliotibial band syndrome (ITBS), prior research has often considered the underlying lateral femoral epicondyle (LFE) as a fixed reference to describe ITB movement during knee flexion, potentially misrepresenting true tissue dynamics. This proof-of-concept study introduces the musculoskeletal advanced transillumination technique (MATT) to visualize and measure LFE displacement relative to the ITB and the tubercule of the ITB (tITB) on the tibia during passive knee flexion. **Methods:** Un-embalmed donor knees (n = 8) were dissected to expose the ITB and positioned on a device allowing standardized passive motion from 0° to 30°. A trocar was inserted between the femoral epicondyles, and a 300-watt xenon light source illuminated the LFE. Video was recorded with an iPhone 15, and key frames were analyzed using ImageJ Version 1.54i, and a custom Python (Version 3.12.5) script to quantify LFE displacement relative to the ITB and to the tITB. **Results:** Median absolute LFE displacement from 0° to 30° was 9.18 mm (IQR 7.23–10.95). Between 0° and 30°, the LFE shifted anteriorly by −1.76 mm (IQR −10.28 to −8.72) relative to the anterior border of the ITB, and by 11.26 mm (IQR 8.27 to 26.33) relative to its posterior border. The LFE-tITB distance increased from 51.98 mm (IQR 49.13–52.36) at 0° to 53.66 mm (IQR 50.08–60.11) at 30°, with a median displacement of 3.92 mm (IQR: 2.48–5.73). **Conclusions:** Musculoskeletal Advance Transillumination Technique (MATT) is a straightforward and reproducible technique that offers direct visualization of the dynamic relationship between a skeletal landmark and myofascial structures, such as the LFE and the ITB. By challenging the assumption that the LFE is a fixed reference point, MATT opens new perspectives for investigating the biomechanical mechanisms underlying conditions like iliotibial band syndrome.

## 1. Introduction

The iliotibial band (ITB) is formed by the union of the aponeuroses of the gluteus maximus and the tensor fascia lata muscles. It extends from the iliac crest and continues onto the linea aspera and the lateral supracondylar line, as the lateral intermuscular septum. In depth, it is attached to the linea aspera and the lateral epicondyle by the proximal and distal Kaplan’s fibers [1]. It finally joins, anteriorly, the lateral patellar retinaculum and, distally, inserts on the tubercle of iliotibial band (tITB) [2]. Some studies describe the ITB as a simple band of fascia, which is more tendinous proximal to the lateral femoral epicondyle (LFE) and ensures lateral knee stability [3], while others highlight its complex structure, consisting of both fascial and muscular components [4]. The dynamic behavior of the ITB is thus influenced by muscular actions and passive tensile forces of connective tissues, making our understanding even more puzzling [2,5]. In the pathokinesiology of iliotibial band syndrome (ITBS), a prevalent overuse injury among runners and cyclists, there is ongoing debate regarding the direction and extent of ITB movement during knee range of motion [6]. There is paucity of functional anatomy studies attempting to elucidate the ITB displacement relative to the LFE during knee flexion–extension movements. Some authors report an antero-posterior translation of the ITB, creating friction on the LFE [7,8]. Combining macro and micro anatomy data with magnetic resonance imaging (MRI) from living humans, Fairclough et al. demonstrated a medial–lateral, rather than anterior–posterior movement, of the ITB about the LFE [3]. However, in another study, a sonographic evaluation confirmed an anterior–posterior movement of the ITB [9].

Neither method fully captures both the movement and the behavior of the fibers. One of the first challenges in studying the relationship between the LFE and the ITB is accurately locating the LFE, which is covered by multiple soft tissue layers. Ultrasound allows visualization of the LFE and the anterior margin of the ITB; however, delineating the posterior margin remains more difficult [9]. Moreover, ultrasound operates in a single imaging plane, which limits its spatial perspective. While MRI provides a more comprehensive anatomical view, it does not allow for dynamic evaluation. This technique relies on segmented static slices acquired over time, preventing a full understanding of the global motion of the structures involved.

There is a critical need to develop approaches that provide a dynamic and anatomically informed perspective on the mechanical behavior of the full width of the ITB relation with the underlying LFE. This is particularly important during knee extension–flexion movements between 0° and 30°, a range at which ITBS symptoms are commonly reported by patients [6,9]. Recent advances in optical tracking and computer vision have demonstrated that light-based point detection methods can offer highly accurate and reproducible measurements of anatomical landmarks, especially when combined with robust filtering and tracking algorithms [10]. The Musculoskeletal Advance Transillumination Technique (MATT) was developed in our laboratory to offer a dynamic and anatomical perspective on the behavior of the full width of ITB in relation to the underlying LFE and its surroundings. This new method enables visualization of the LFE displacement under the intact ITB by transilluminating the LFE from its inner surface using an arthroscopic light source during passive, standardized 0° to 30° knee extension–flexion movements applied to a fresh cadaver’s sample.

The objective of the present study is to prove the feasibility and application of a novel approach based on light transillumination of the LFE using an arthroscopic xenon light source to visualize and measure movements of a deep bony structure over intact overlying soft tissue envelope.

## 2. Materials and Methods

### 2.1. Study Design

This is a descriptive proof-of-concept study conducted in an anatomy laboratory, designed to assess the feasibility of the MATT. Knees from fresh cadavers were used for the purpose of this study. Donors gave informed consent for their bodies to be used for scientific research and teaching purposes at the University of Sherbrooke under the Public Health Protection Act established by the Quebec government. Donors with knee deformity, such as surgical or traumatic scars or a rigid flexum, were excluded.

### 2.2. A Step-by-Step Protocol for the Musculoskeletal Advanced Transillumination Technique (MATT) for Cadaveric Specimens

**Figure 1:**
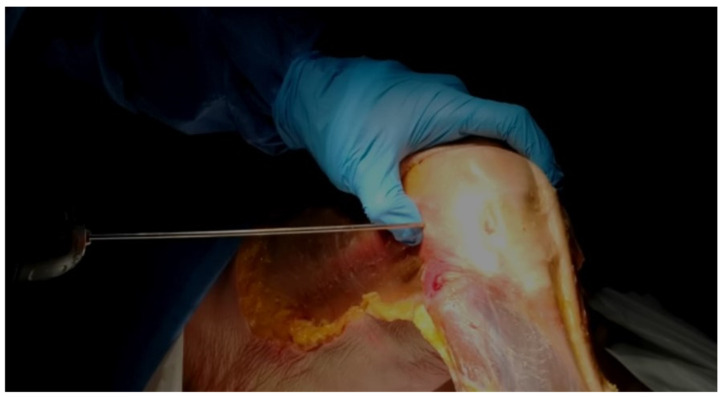
Wire Insertion at the LFE. Direct palpation of the LFE and insertion of the 2 mm wire at its apex, aiming medially.

**Figure 2:**
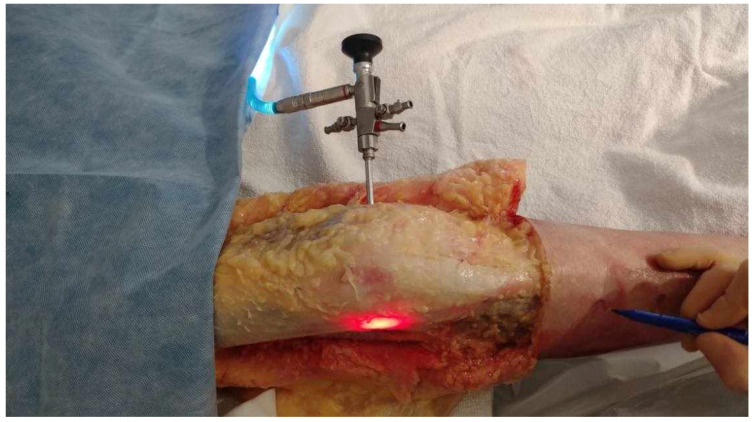
Medial to lateral insertion of the scope. Frontal view of the knee. The initial 2 mm pin track is first dilated from medial to lateral with the 2.9 mm obturator and then with the (5.2 mm) arthroscope sheath. The scope is advanced until its tip is in contact with the inner cortex of the LFE. A 300-watt Xenon light source is connected to the arthroscope at 25% of its maximum power to transilluminate the LFE under the ITB.

**Figure 3:**
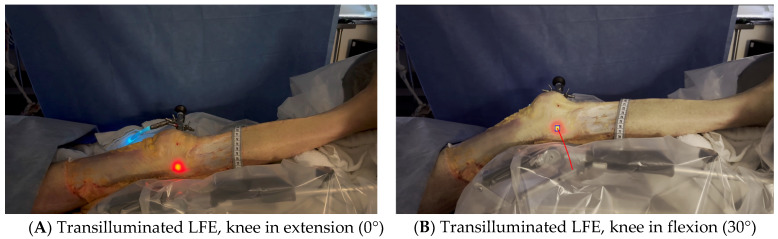
Transilluminated LFE. Experimental setup with the leg positioned on the Kinetec Optima Continuous Passive Motion (CPM) device. The absolute displacement of the light-defined point corresponding to the LFE can be observed between position (**A**) (0°) and position (**B**) (30°) of knee flexion, enabling precise measurement of its movement. Red line in B shows absolute displacement of the LFE from 0° to 30°.

**Figure 4:**
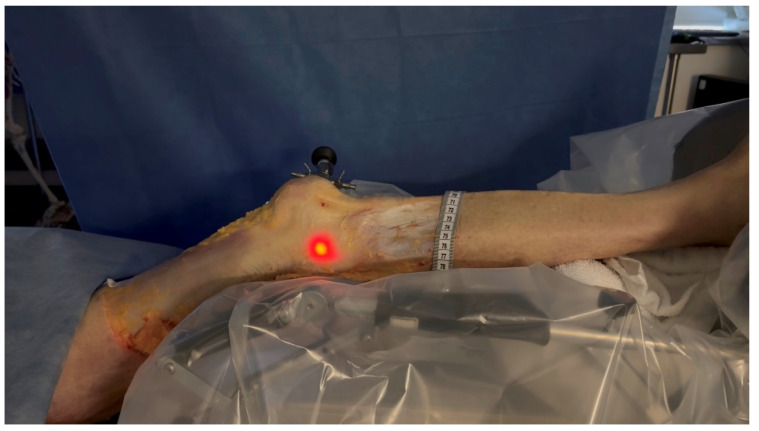
Leg positioned in the CPM. The leg was positioned in a CPM system to control the extension to flexion movement from 0° to 30°.

1. Dissection of the anterior and lateral region of the knee was carried out by one orthopedic surgeon using the same protocol for each case. He proceeded by incising a large rectangular skin flap centered on the lateral aspect of the thigh, from the femoral middle 1/3 to the tibial proximal 1/3. The knee extensor apparatus, ITB, and distal lateral hamstring insertion were exposed and cleaned of subcutaneous fatty tissue. The tITB was identified and exposed.
2. The LFE was located by palpation through the ITB layer by the orthopedic surgeon.
3. Using the tip of the LFE as the entry point, a 2 mm wire was inserted with a drill into the LFE from lateral to medial through the ITB. This created a transosseous femoral tunnel while preserving the structural integrity of the ITB. (Figure 1)
4. The exit point on the medial femoral condyle was carefully identified and exposed. The wire was then completely removed from the medial side.
5. A 2.9 mm conical arthroscopic obturator (T4902, Conmed, Clearwater, FL, USA) was used as a dilator. It was introduced into the medial femoral condyle exit hole previously created by the 2 mm wire. The obturator was then tapped in with gentle mallet blows until its tip barely emerged from the LFE as felt by palpation.
6. The obturator was then removed and replaced by a 5.2 mm double-valve high-flow arthroscope sleeve (T4932, Conmed, Clearwater, FL, USA), compatible with a 2.9 mm, 30° arthroscope (T2930, Conmed, Clearwater, FL, USA), loaded with its obturator. The sleeve was then gently advanced with back-and-forth rotational movements until the tip was in contact with the inner cortex of the LFE (Figure 2). After replacement of the obturator by the 30° arthroscopic lens, a 300-watt Xenon light source (LS8000, Conmed, Clearwater, FL, USA) was connected.
7. Light was projected at 25% maximum intensity onto the inner cortex of the LFE. A transilluminated area became clearly visible through the overlying fibers of the fascia lata. The transilluminated zone, referred to as visual landmark point A and point B, was subsequently identified and marked on the recorded images in both positions of interest at 0° and 30° knee flexion, respectively (Figure 3).
8. The leg was then placed on a Kinetec Optima knee Continuous Passive Motion (CPM) device (S & U Medizintechnik GmbH, Zotzenheim, Germany), which allowed standardized passive knee extension–flexion cycles ranging from 0° to 30° at a constant angular velocity of 3° per second. Patellar orientation was maintained horizontally to ensure neutral rotation, and no straps were used to preserve unrestricted hip motion in both sagittal and coronal planes (Figure 4).

### 2.3. Trocar Placement Validation

In order to validate the position of the light source in relation to the tip of the LFE, a radiologic imagery modality was used. Fluoroscopy was initially planned for all the procedures, but a last-minute technical failure prevented us from using it for the first 4 knees. A backup option was then to use a research-dedicated CT scan after the procedure for those knees.

A CT scan was therefore performed on donors #1 and #2 (4 knees) to validate wire positioning and ensure proper alignment with the inner cortex of the apex of the LFE after the procedures. For donors #3 and #4 (4 knees), a fluoroscopy image intensifier device (C-arm) was available and used on both knees to visually assess wire placement at the apex of the LFE during procedures, both before drilling and after insertion. Unlike the CT scan, which provides retrospective evaluation, fluoroscopy enables real-time visualization of anatomical structures during the procedure and is more cost-effective. Wire placement was then compared between blind drilling and fluoroscopy-assisted drilling.

### 2.4. Image Acquisition and Post-Processing

Knee flexion movements from 0 to 30° were recorded using an iPhone 15 camera mounted on a stationary tripod to ensure stable and consistent framing throughout the intervention. The camera features a 48-megapixel sensor with an f/1.6 aperture and sensor-shift optical image stabilization. A millimetric ruler was put next to the tITB for length calibration purposes. The tripod height was adjusted so that the camera lens was aligned horizontally with the center of the lateral femoral epicondyle, and the angle of incidence was kept perpendicular to the sagittal plane of the knee throughout the recording. These measures were taken to ensure consistent framing and minimize perspective distortion.

The configuration and alignment of the arthroscope were standardized for all measurements. A 300 W xenon light source was operated at 25% intensity to minimize glare and reduce light scattering through the fascial tissues. The distance between the arthroscope and the exterior surface was kept minimal. Calibration procedures included a minor angulation correction. Potential sources of systematic error, such as variability in fascial thickness—which could influence light dispersion and centroid detection—were considered. Light dispersion was further evaluated using Monte Carlo simulations implemented in PyTissueOptics (DCC-Lab, CERVO Brain Research Center, Québec, Canada), based on the model described by Wang et al. (1995) [11].

**Figure 5:**
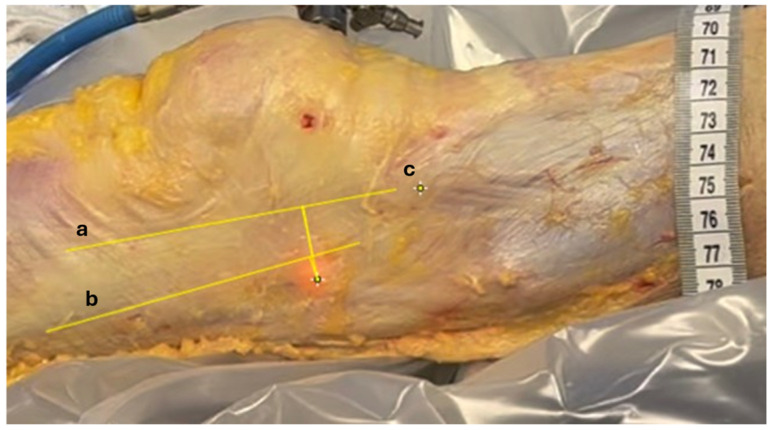
Measuring relative displacements. Anatomic structures identified on recorded images. Longitudinal yellow lines between (a) anterior margin of ITB, (b) posterior margin of ITB, and (c) tITB. The distance between the transilluminated LFE at 0° and 30° of knee flexion and these 3 landmarks were measured using video footage recorded during the procedure.

Distance measurements were computed using ImageJ, Version 1.54i, U.S. National Institutes of Health, Bethesda, MD, USA. A millimetric ruler was placed adjacent to the tITB during video capture. This allowed us to calibrate the scale of the image by converting the number of pixels corresponding to 1 cm on the ruler, enabling accurate distance measurements in the recorded footage. The transilluminated projection of the LFE was identified at full extension (point A) and at 30° of knee flexion (point B), based on the centroid of the illuminated region visible through the fascia lata. The anterior and posterior borders of the ITB, as well as the position of the tITB, were manually annotated by the orthopedic surgeon on the recorded images using visible anatomical landmarks (Figure 5).

**Figure 6:**
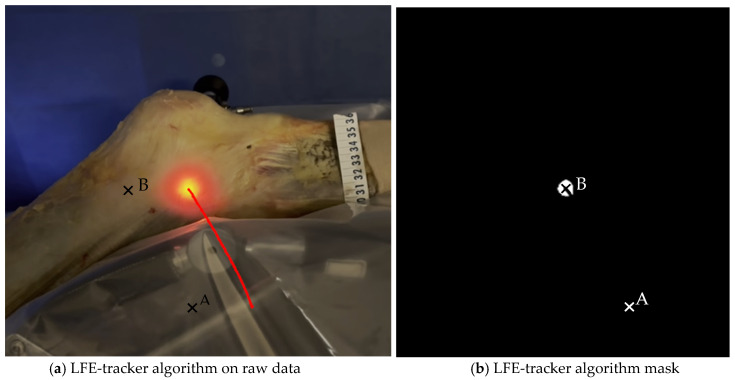
Image analysis algorithm. (**a**) The centroid of the light-defined area corresponding to the LFE is identified using a custom Python script that analyzes pixel intensity and the geometric properties of the illuminated region. The red line represents the trajectory of the light hollow throughout the movement. (**b**) This panel shows the result after applying an intensity filter. A millimetric ruler is used for calibration.

A custom Python algorithm (version 3.12.5, Python Software Foundation, Wilmington, DE, USA) was then applied to detect and outline the illuminated area based on light intensity and geometric properties. It automatically computed the centroid coordinates of the LFE in both positions and calculated linear displacements in the sagittal plane. These displacements were quantified (1) between point A and point B to evaluate global LFE movement and (2) relative to the ITB anterior and posterior borders and the tITB at each timepoint. This process enabled both absolute and relative measurements of LFE displacement during passive knee motion (Figure 6). The code is openly available in a repository and can be found in the Supplementary Materials.

### 2.5. Identification of Anatomical Landmarks

**Figure 7:**
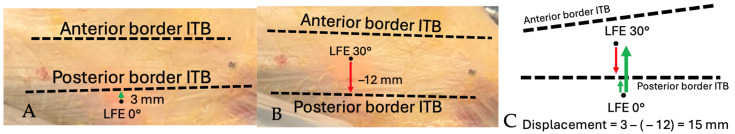
Schematic demonstration of displacement measurement technique: Distance from LFE to posterior border of ITB. (**A**) Distance measured from LFE to posterior border of ITB at 0°. Positive value from posterior to anterior. (**B**) Distance measured from LFE to posterior border of ITB at 30°. Negative value if LFE crossed over posterior border of ITB. (**C**) Total anterior displacement is measured by subtracting the distance value at 30° from the distance at 0°. Thick arrow shows the total displacement.

**Figure 8:**
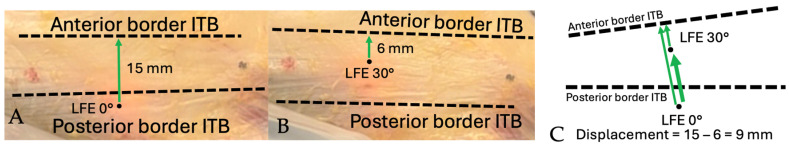
Schematic demonstration of displacement measurement technique: Distance from LFE to anterior border of ITB. (**A**) Distance measured from LFE to anterior border of ITB at 0°. Positive value from posterior to anterior. (**B**) Distance measured from LFE to anterior border of ITB at 30°. Positive value from posterior to anterior. (**C**) Total anterior displacement is measured by subtracting the distance value at 30° from the distance at 0°. Thick arrow shows the total displacement.

The anterior and posterior borders of the ITB, as well as the position of the tITB, were manually identified by the orthopedic surgeon on the recorded video sequences. Landmark identification was performed frame-by-frame using anatomical continuity of the fascia lata and visual landmarks observed intraoperatively. These reference points were annotated within ImageJ and served as the basis for calculating the spatial relationship between the transilluminated LFE and surrounding structures during the movement cycle. Distances were first measured between the LFE and the anterior and posterior ITB borders at 0° and subsequently at 30° of knee flexion. When the LFE crossed involved the ITB border between 0° and 30°, the measurement was expressed as a negative value. Figure 7 and Figure 8 illustrate the method used to measure the displacement of the LFE relative to the ITB between 0° and 30° of flexion.

### 2.6. Data Analyses

The position and distance between the LFE and tITB were summarized using the median and interquartile range (IQR). The Python algorithm processes each frame to detect, track, and analyze the movement and characteristics of the light hollow emitted by the light source. It efficiently identifies the light hollow, outlines its contour, computes its centroid and bounding box, and displays these elements in real time. The methodology ensures accurate and consistent detection as well as a clear visualization of the structure of interest.

## 3. Results

The procedure was conducted on both knees of four donors (three males and one female) totaling eight knees. The median age of death was 73.5 years (IQR 71.25–75.25). The demographic data are reported in Table 1.

The LFE exhibited a median absolute sagittal displacement of 9.18 mm (anterior) between 0° and 30° of passive knee flexion.

With increasing knee flexion from 0° to 30°, the lateral femoral epicondyle (LFE) shifted anteriorly, relative to the iliotibial band (ITB). The median distance to the posterior ITB margin changed from 2.72 mm (IQR −8.80–5.95) at 0° to −6.23 mm (IQR −11.77–11.49) at 30°, for a total relative anterior displacement of 8.95 mm. The distance to the anterior margin decreased from 13.24 mm (IQR 8.84–22.10) to 9.29 mm (IQR 7.69–30.58) for a total relative anterior displacement of 3.95 mm.

A progressive increase in the LFE-to-tITB distance was also observed from 0° to 30°, with a median change of 3.92 mm. Complete measurement data are provided in Table 2.

### Trocar Placement Validation

**Figure 9:**
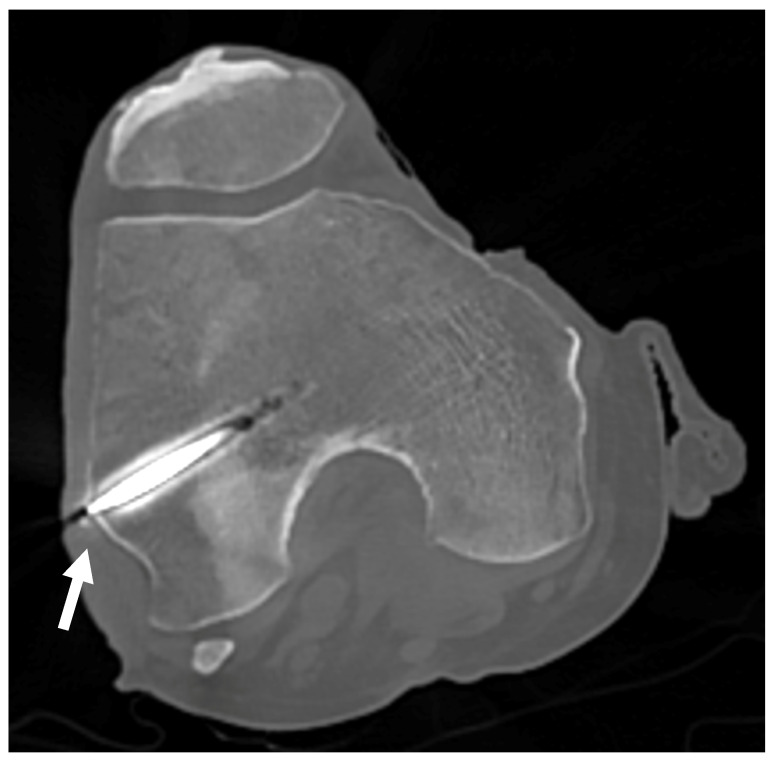
CT scan of knee 1. CT scan used to confirm proper positioning of the trocar for knees 1 to 4. The white arrow points to the LFE, confirming alignment with the targeted anatomical structure.

In the first set of samples (knees 1 to 4), the trocars were correctly placed towards the LFE, except for the left leg of knee 1, which showed a distance of 6.7 mm between the tip of the wire and the apex of the LFE as measured on the CT scan coronal view (Figure 9). The results are reported in Table 3.

**Figure 10:**
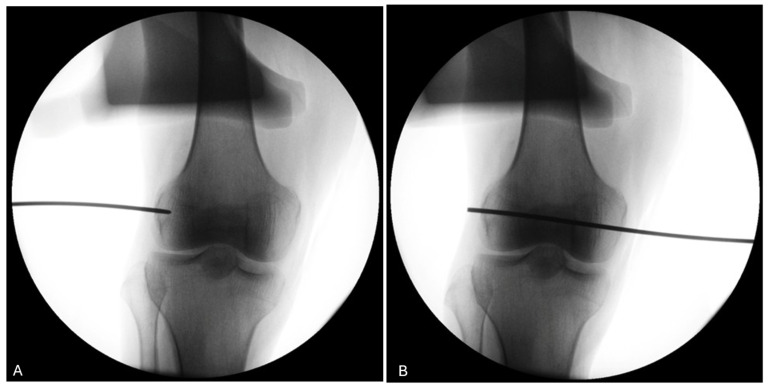
Fluoroscopy of the right knee of donor #4. (**A**) The use of fluoroscopy on a right knee (donor #4) confirms that the pin entry point (black line) is well-centered visually on the LFE on the antero-posterior view. (**B**) The pin is advanced medially to exit through the medial condyle.

The use of a fluoroscopy device during the procedure (knees 5 to 8) demonstrates its utility in ensuring the proper positioning of the trocar for the four knees of donor #3 and #4 (Figure 10).

## 4. Discussion

This proof-of-concept study aims at describing the potential of the Musculoskeletal Advanced Transillumination Technique (MATT) developed in our laboratory. MATT enabled direct visualization and quantification of the LFE displacement beneath the ITB in the sagittal plane, as well as the relative displacement between the LFE and the tITB. Inexpensive motion quantification methods were assessed to enhance usability and to improve understanding of the dynamic relationship between these structures.

While prior studies have commonly assessed ITB displacement relative to the LFE—implicitly treating the LFE as a fixed anatomical landmark—our results suggest that this assumption may be suboptimal [6,8,9]. Specifically, we observed a measurable displacement of the LFE relative to the Gerdy’s tubercle of 3.92 mm between 0° and 30° of knee flexion. These finding highlights that the LFE may not be a static structure, but rather one that moves during early flexion. These insights, although not directly linked to clinical or functional outcomes in this study, may help refine our biomechanical understanding of ITB-LFE interactions. They also suggest that friction between the ITB and the LFE may not stem solely from ITB translation over a fixed bony prominence, as previously proposed.

In a previous study, Jelsing et al. [9] used sonographic assessment to investigate ITB displacement and reported a 3.8 mm posterior translation of the anterior ITB fibers relative to the LFE between 0° and 45° of knee flexion. In our study, we observed that the LFE moved anteriorly by 3.95 mm relative to the anterior border of the ITB. The direction and magnitude of this displacement are comparable with those reported by Jelsing et al. However, one limitation of our methodology is that ultrasonography was not performed prior to dissection, which would have allowed direct comparison of the same specimens and further strengthened the validity of our findings. Future studies should consider integrating ultrasound as a complementary, non-invasive modality to validate displacement tracking prior to anatomical exposure.

Interestingly, when using the posterior border of the ITB as a reference, the LFE was found to move anteriorly by 8.95 mm. This larger displacement could reflect ITB deformation during motion or result from the greater difficulty in accurately delineating the posterior edge—an issue also reported by other authors [8].

Although this study serves as a proof of concept, further research using the MATT system will be needed to precisely quantify the respective displacements of the LFE and ITB in multiple planes. Such investigations could provide deeper insight into the pathomechanics of iliotibial band syndrome (ITBS), particularly in the context of repetitive flexion–extension activities such as running or cycling.

### Limitations

As this was a proof-of-concept study with a small sample size, our aim was not to generalize the findings, but rather to demonstrate the feasibility and potential application of the MATT. No statistical generalization can be drawn from our results.

Although only non-embalmed, fresh bodies were included to ensure optimal tissue conditions for demonstrating the concept that the ITB and LFE can be visualized and tracked with the MATT, hip and knee joint range of motions were not documented. However, all hips and knees could be passively mobilized within the range of interest (0° to 30° of flexion). We acknowledge that the resistance to flexion was not quantified, which may have influenced the mechanical properties of the ITB. Nevertheless, while such factors could potentially affect the fascia lata’s mechanical properties, they did not impair the ability of the MATT to achieve transillumination or displacement tracking of the anatomical structures of interest. Future studies could further explore how such histological or biomechanical alterations may influence MATT-based measurements.

No formal intra operator reliability assessment was conducted however, guide wire positioning was retrospectively verified. In the first four knees, CT imaging was used retrospectively to verify trocar positioning due to an unexpected failure of the fluoroscopy unit. Once fluoroscopy was operational, it was used intraoperatively for the remaining knees. The use of both imaging modalities ultimately reinforces the applicability of our protocol, as it demonstrates that trocar positioning can be reliably assessed using either CT or fluoroscopy, depending on equipment availability in laboratory settings. All procedures were performed by a fellowship-trained orthopedic surgeon specializing in sports medicine. In one of the CT-evaluated knees, a misalignment greater than 6 mm was observed. This was on the first specimen, and the surgeon indeed noted a slight glide of the tip of the pin at insertion. A more careful insertion technique was then used to avoid the same mistake. However, the light-defined point used for measurements remained stable, and the average relative displacement between 0° and 30° of flexion was therefore unaffected. The use of fluoroscopy gives direct inputs on proper pin placement on the antero-posterior (AP) view and may be a less expensive technique. On the other hand, since the LFE is impossible to locate from the lateral view, it leaves only the AP view to locate the projection of the contour of the LFE. This may lead to undetected anterior or posterior placement of the wire. Both techniques therefore have their limitations. The best scenario would be to insert the wire, use the CT scan to validate the proper position, and then bring the cadaveric knee back to the lab to move on with the measurements. In our setting, this was not practicable.

Another limitation of the present study is that anatomical landmarks were manually annotated by a single operator, and no inter- or intra-rater reliability analysis was performed. Future studies should include reproducibility assessments to strengthen the methodological robustness of MATT-based measurements.

Ideally, a 0° arthroscope should be used to be sure that the centroid of the light-defined point is in a straight line with the tip of the scope. Unfortunately, only a 30° scope was available in our lab. To mitigate this limitation, we standardized the setup by orienting the 30° lens distally in all specimens. This consistent positioning reduced the potential for angular variability between trials. Additionally, since our goal was to assess relative displacement between structures rather than absolute positional coordinates, we believe this limitation did not compromise the feasibility assessment or the relative motion measurements. Importantly, the arthroscope remained static throughout the experiment, anchored in the same epicondylar position, which ensured that any observed motion of the LFE was independent of the scope’s viewing angle.

Nonetheless, MATT could have the potential to study other frictional and snapping syndromes of the musculoskeletal system, such as snapping hip syndrome [12], greater trochanteric pain syndrome [13], snapping elbow syndrome [14], snapping triceps syndrome [15], snapping wrist [16,17], snapping pes anserinus [18,19], snapping biceps femoris syndrome [20], and snapping popliteus tendon syndrome [21].

## 5. Conclusions

MATT is a straightforward, step-by-step technique that could help researchers gain a deeper understanding of how skeletal anatomy behaves in relation to surrounding myofascial tissues, such as the LFE in relation to the ITB. Beyond providing clear visual access to deep anatomical layers, MATT also enables direct observation of dynamic interactions between structures previously assumed to be fixed or passive. This technique holds promise for re-evaluating long-standing biomechanical models—such as the role of ITB friction over a static LFE—and could contribute to a more accurate understanding of the pathomechanics involved in conditions like iliotibial band syndrome.

## Figures and Tables

**Table 1:** Demographic information of donors.

Donors	1	2	3	4
Age (years)	76	75	72	69
Sex	F	M	M	M
Time since death (days)	47	7	25	27

**Table 2:** Lateral femoral epicondyle (LFE) displacement measurements.

	N	Minimum	Maximum	Median	IQR
Absolute LFE displacement from 0° to 30° (mm)	8	1.94	14.95	9.18	7.23–10.95
Distance between LFE and posterior margin ITB at 0° (mm)	8	−12.32	13.39	2.72	−8.80–5.95
Distance between LFE and posterior margin ITB at 30° (mm)	8	−15.27	19.13	−6.23	−11.77–11.49
Mean change between LFE and posterior margin ITB (mm)	8	−13.8	16.26	−1.76	−10.28–8.72
Distance between LFE and anterior margin ITB at 0° (mm)	8	7.50	28.20	13.24	8.84–22.10
Distance between LFE and anterior margin ITB at 30° (mm)	8	5.73	35.32	9.29	7.69–30.58
Mean change between LFE and anterior margin ITB (mm)	8	6.61	31.76	11.26	8.27–26.33
Distance between LFE and tITB at 0° (mm)	8	39.71	55.83	51.98	49.13–52.36
Distance between LFE and tITB at 30° (mm)	8	45.44	63.67	53.66	50.08–60.11
Mean change in LFE–tITB distance during knee flexion from 0° and 30° (mm)	8	1.64	8.08	3.92	2.48–5.73

**Table 3:** Distance between the tip of the trocar and the apex of LFE, measured by CT scan.

Donor #1 (mm)		Donor #2 (mm)	
Left	Right	Left	Right
6.7	0.7	1.1	1.6

## Data Availability

The original dataset and the Python algorithm presented in the study are openly available in Zenodo at https://doi.org/10.5281/zenodo.16875886; accessed on 14 August 2025.

## Supplementary Materials

The following supporting information can be downloaded at: https://github.com/MedCodeUdeM/MATT_CHUS, accessed on 14 August 2025.

## Abbreviations

The following abbreviations are used in this manuscript:

MATT	Musculoskeletal Advanced Transillumination Technique
ITB	Iliotibial Band
ITBS	Iliotibial Band Syndrome
LFE	Lateral Femoral Epicondyle
tITB	Tubercule of the Iliotibial Band
MRI	Magnetic Resonance Imaging
CPM	Continuous Passive Motion
CT Scan	Computerized Tomography Scan
C-arm	Fluoroscopy Image Intensifier device
IQR	Interquartile Range
AP	Antero-posterior
